# Pregnancy in a Patient With Sick Sinus Syndrome and a Permanent Pacemaker

**DOI:** 10.7759/cureus.90289

**Published:** 2025-08-17

**Authors:** Nimmi Varghese, Lakmini Malika Adikari

**Affiliations:** 1 Obstetrics and Gynaecology, Stoke Mandeville Hospital, Aylesbury, GBR

**Keywords:** arrhythmias, beta blocker, fetal growth, pacemaker, pregnancy, sick sinus syndrome

## Abstract

Sick sinus syndrome (SSS) is a disorder of the sinoatrial node characterised by impaired pacemaker function and abnormal impulse transmission, resulting in arrhythmias. Definitive treatment involves replacing the defective pacemaker with a synthetic one. With well-functioning pacemaker support, patients with SSS can achieve successful pregnancy outcomes. Here, we are reporting a case of a 29-year-old woman in her first pregnancy, who was diagnosed with SSS. Three years back, she was evaluated for this condition in view of her history of recurrent blackouts. She underwent permanent pacemaker implantation, which significantly improved her symptoms, and she was maintained on bisoprolol. Her pregnancy was managed jointly with the cardiology team, and her pacemaker function was closely monitored. Fetal growth was monitored using serial scans and was within normal limits. She went into spontaneous labor at 39 weeks of gestation, received epidural analgesia for pain relief, and had a successful vaginal delivery without any complications.

## Introduction

Sick sinus syndrome (SSS) refers to a group of cardiac arrhythmias wherein the functioning of the sinoatrial node is severely impaired, leading to abnormalities in cardiac rhythm and conduction. The pathogenesis for such implications has not been clearly demonstrated. The occurrence of this condition in healthy people without any evident structural heart disease has also been reported [1].

The common rhythm abnormalities include atrial bradyarrhythmias, tachyarrhythmias, and occasionally a combination of bradycardia and tachycardia. These arrhythmias may manifest as palpitations, fatigue, lightheadedness, presyncope, and syncope due to decreased tissue perfusion. The recommended definitive treatment is to replace the defective pacemaker with a synthetic one [2].

With a well-functioning pacemaker, patients with SSS can achieve a successful pregnancy outcome. However, pregnancy may exacerbate symptoms such as palpitations, dizziness and fatigue, and in severe cases of SSS, complications like preterm labour have also been reported [2].

## Case presentation

A 29-year-old woman with a known diagnosis of SSS was managed in our hospital for her pregnancy care during her first pregnancy. She was evaluated for recurrent blackouts three years prior to conception, and the diagnosis of SSS was reached upon.

**Figure 1:**
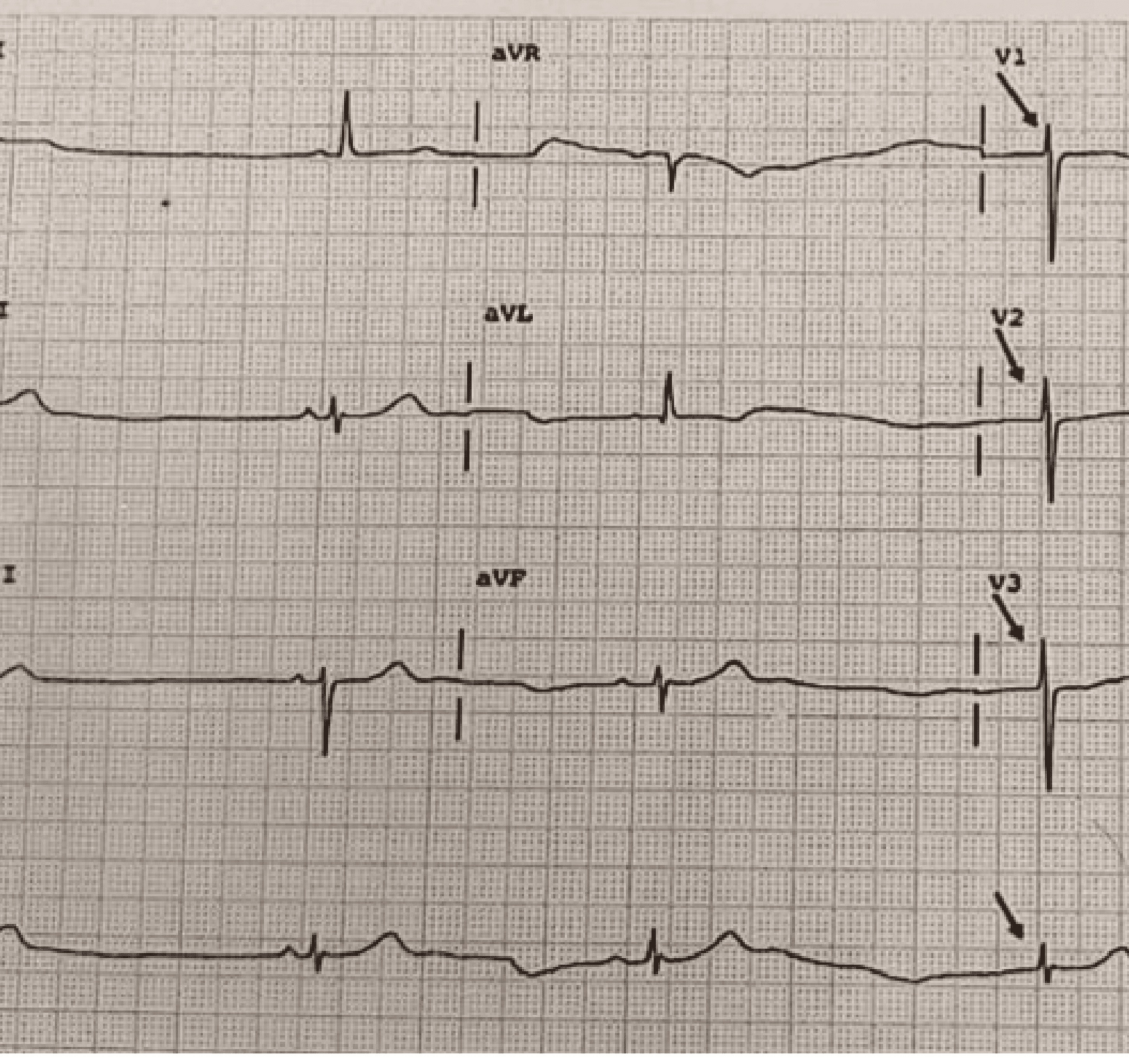
ECG of the patient at the time of diagnosis

The ECG below shows sinus bradycardia at 37 beats per minute with junctional rhythm in between (Figure 1). There is a normal P wave with PR interval being 120 ms, narrow QRS, normal QT interval with no ST-T changes. This was suggestive of SSS.

She underwent permanent pacemaker implantation, which significantly improved her symptoms. She was maintained on cardio selective beta-blocker therapy with bisoprolol, and her pacemaker function was closely monitored. 

During her pregnancy, she remained under close follow-up with cardiologists. While she experienced occasional break through palpitations, her symptoms were otherwise well-controlled. She had regular pacemaker follow-up, and ECG was done at recommended intervals as per the cardiology plan. Bisoprolol therapy was continued throughout her pregnancy, and serial foetal growth scans were performed to monitor for potential growth restriction due to beta-blocker use. The foetal growth remained within normal limits in the scans. 

At 39 weeks of gestation, she went into spontaneous labour. Epidural analgesia was provided for pain relief, and she had a successful vaginal delivery without complications. Her baby’s growth was appropriate for her gestational age and had good APGAR scores. Her postpartum period was uneventful and was discharged home as per routine care plan. She will continue to be under cardiology follow-up and will follow the same pattern as in her pre-pregnancy plan.

## Discussion

SSS is a generalised abnormality of cardiac impulse formation wherein the heart's natural pacemaker is damaged and is not able to perform its normal pace-making function. There can be both intrinsic and extrinsic reasons for this condition [3].

Pregnancy is a physiological state in which the blood flow is maintained on a higher side keeping the resistance to flow to a bare minimum to cater to the needs of the growing baby. There are progressive cardiovascular accommodations occurring in each trimester that can persist into the postpartum period as well. It has been observed that in patients with a pre-existing cardiac condition or in those who develop a cardiovascular abnormality during pregnancy, the risk of morbidity to the mother and fetus is more [4]. Studies show that only 1-4% of the pregnancies are challenged by a heart condition in the mother and most of these can be managed successfully with a favourable outcome in the pregnancy and postpartum [5].

The haemodynamic changes occurring during pregnancy include an increase in blood volume, cardiac output, and stroke volume, leading to increase in preload and an increase in heart rate causing pulmonary oedema. These physiological changes that occur in every normal pregnancy can put added stress on a mother with an underlying cardiac condition, thus leading to several complications that may arise during the antenatal, intrapartum, or postnatal period [2].

The mechanisms leading to impaired function of the sinoatrial node in SSS patients remains vague. Degenerative fibrosis of the nodal tissue has been the closest reported reason in the literature [2]. Some of the newer studies have found out certain gene mutations, including the SCN5A gene, in congenital SSS patients [1].

SSS can be asymptomatic or mildly symptomatic with non-specific symptoms like bradyarrhythmia, tachyarrhythmia, loss of consciousness, palpitation, and feeling dizzy, and the diagnosis of the condition can be quite tricky. Hence, it is important to perform a thorough physical examination along with an electrocardiogram, including performing the carotid sinus pressure, while observing the electrocardiogram. An asystole response of three or more seconds while applying pressure on the carotid sinus is a strong predictor for the diagnosis of SSS and one of the criteria for inserting a permanent pacemaker if the patient has a history of syncope [6]. A 24-hour Holter monitoring and echocardiogram will further aid in the management of the condition. Common findings on the ECG include sinus bradycardia, sinus pauses, junctional escape rhythms, sinoatrial (SA) exit block, and alternating periods of tachycardia and bradycardia.

It is important to have a multidisciplinary approach to manage this condition involving the obstetrician, cardiologist, and an anaesthetist trained in high-risk obstetrics. However, this does not mean that the input from the obstetric side should be limited. It is very essential to involve a senior obstetrician trained in high-risk obstetrics to manage the complexities of the situation [7]. Close monitoring, including regular assessment of pacemaker function, forms the primary pillar of care. Pregnancy-related symptoms may be exacerbated due to the underlying condition. Arrhythmias are managed by beta-blockers like bisoprolol, which has minimal protein binding capacity and moderate lipid solubility. It has been proven to have a good oral bioavailability and a longer t1/2. Although the excretion amount through the kidneys is high, its potential to accumulate in the baby should be considered and hence should be used with adequate caution [8]. The potential impact on foetal growth should be considered, and serial growth scans help in ruling out this concern as part of antenatal care [2].

Complications that may arise include acute pulmonary oedema, infective endocarditis, congestive cardiac failure, aortic dissection, and pulmonary hypertension [2].

Replacement with a prosthetic pacemaker is the definite treatment option [2]. Pacemakers do not reduce mortality, but they can decrease symptoms and improve the quality of life [9]. It must be kept in mind that the implantation of the pacemaker during the period of foetal organogenesis should be avoided. Reports suggest that the use of reversed image fluoroscopy and a toco-monitor can help with a smooth and safe pacemaker implantation [10].

The mode of delivery should be guided by obstetric indications, with no special requirements for labour in most cases. However, for patients undergoing caesarean section, bipolar diathermy is recommended to minimize interference with pacemaker function. The electrosurgical receiving plate must be positioned in such a way that the current pathway does not pass through or near the pacemaker. Moreover, it is important to restrict the electrocautery bursts to one second for every 10-second interval [11].

## Conclusions

SSS represents a spectrum of sinoatrial node dysfunction that often manifests with symptomatic arrythmia patterns. Although pharmacological management can provide benefits in managing certain symptoms, the cornerstone for treatment remains permanent pacemaker implantation. With appropriate multidisciplinary input and symptom control, patients with SSS and a pacemaker can achieve favourable pregnancy outcomes. Future research should continue to explore individualised approach to patient selection to further refine outcomes in this population.
